# Characterization of the chloroplast genome of a rare species in China, *Lilium martagon* var. *pilosiusculum*

**DOI:** 10.1080/23802359.2018.1450667

**Published:** 2018-04-01

**Authors:** Yanping Zhou, Yu Bi, Qiheng Yan, Guixia Jia, Yunpeng Du

**Affiliations:** a Beijing Key Laboratory of Ornamental Plants Germplasm Innovation & Molecular Breeding, National Engineering Research Center for Floriculture and College of Landscape Architecture, Beijing Forestry University, Beijing, China;; b Beijing Agro-Biotechnology Research Center, Beijing Academy of Agriculture and Forestry Sciences, Beijing, China;; c Beijing Key Laboratory of Agricultural Genetic Resources and Biotechnology, Beijing, China;; d Biological Sciences, University of California, Irvine, America

**Keywords:** *Lilium martagon* var. *pilosiusculum*, high-throughput sequencing, chloroplast, genome sequence

## Abstract

*Lilium martagon* var. *pilosiusculum* is an endangered species with high ornamental value in Xinjiang province (China). In this study, we reported a complete chloroplast genome of *L. martagon* var. *pilosiusculum*, which was *de novo* assembled using the next-generation sequencing data. The complete chloroplast genome is 152,816 in length, including a large single copy region of 82,265 bp and a small single copy region of 17,541 bp and two inverted repeat regions of 26,505 bp. A total of 110 functional genes were encoded, consisting of 76 protein-coding genes, 30 transfer RNA genes, and four ribosomal RNA genes. The overall AT content of the chloroplast genome is 63.00%. In addition, 68 SSRs and 31 large repeat sequences were found. In the maximum likelihood tree, a strong phylogenetic signal showed that *L. martagon* var. *pilosiusculum* is a species of *lilium*.

*Lilium martagon* Linnaeus var. *pilosiusculum* Freyn is a perennial flower bulbs belonging to the section *Martagon*, the genus *Lilium* (Liliaceae). *L. martagon* var. *pilosiusculum* is characterized by leaves whorled, flowers 2–7 in a raceme, nodding to horizontal, tepals purple-red with deeply coloured spots, nectaries papillose on both surfaces (Liang and Tamura 2000). It is a popular ornamental. In China, this species is only distributed in Xinjiang province. However, it is considered as a rare and endangered species in Information System of Chinese Rare and Endangered Plants (ISCREP) (http://rep.iplant.cn/prot/Lilium%20martagon%20var.%20pilosiusculum) and China Biodiversity Red List: Higher Plants (http://www.zhb.gov.cn/gkml/hbb/bgg/201309/t20130912_260061.htm) due to the human activities, unreasonable deforestation and habitat deterioration. Based on the complete sequencing of chloroplast genome, the genetic background can be better understood, which is helpful in promoting the preservation of the species.

A wild individual of *L. martagon* var. *pilosiusculum* was collected from Xinjiang province, China. Voucher specimen was deposited in Beijing Agro-Biotechnology Research Center (Beijing, China). Total genomic DNA was extracted from fresh leaves, according to the DNAsecure Plant Kit (Aidlab). A genomic DNA library was constructed using VAHTSTM Turbo DNA Library Prep Kit for Illumina^®^ (Vazyme, Nanjing City, China). High-throughput sequencing was performed with pair-end reads on the HiSeq4000 Sequencing System at Novogene (http://www.novogene.com/index.php). The raw reads were quality-trimmed by NGSQC Toolkit v2.3.3 (Patel and Jain 2012) and assembled by SPAdes v3.6.1 (Bankevich et al. 2012). Assembled chloroplast genome was annotated using Dual Organellar GenoMe Annotator (http://dogma.ccbb.utexas.edu/) (Wyman et al. 2004). The gene map of the cp genome was drawn in OGDraw v1.2 (Lohse et al. 2013).

Whole chloroplast genome sequence of *L. martagon* var. *pilosiusculum* has been submitted to GenBank with the accession number MF964219. It is 152,816 in length, including a large single copy (LSC) region of 82,265 bp and a small single copy (SSC) region of 17,541 bp and two inverted repeat (IR) regions of 26,505 bp. The complete cp-DNA encodes 110 genes, comprising 76 protein-coding genes, 30 transfer RNA genes, and four ribosomal RNA genes. Among these genes, 15 genes (*trnA-UGC*, *trnG-GCC*, *trnI-GAU*, *trnK-UUU*, *trnL-UAA*, *trnV-UAC*, *rps16*, *petD*, *atpF*, *rpl16*, *petB*, *rpl2*, *ndhA*, *ndhB*, and *rpoC1*) contained one intron, two genes (*ycf3* and *clpP*) contained two introns and 19 genes (*rrn4.5*, *rrn5*, *rrn16*, *rrn23*, *trnA-UGC*, *trnH-GUG*, *trnI-CAU*, *trnI-GAU*, *trnL-CAA*, *trnN-GUU*, *trnR-ACG*, *trnV-GAC*, *ndhB*, *rps7*, *rpl2*, *rpl23*, *ycf2*, *ycf1*, and *rps19*) were located in IR region. The nucleotide composition of *L. martagon* var. *pilosiusculum* has high A + T content of 63.00%, and the corresponding values of the SSC, LSC, and IR regions were 69.30%, 65.20%, and 57.60%, respectively.

The perl program MISA (http://pgrc.ipk-gatersleben.de/misa/) was used to identify the simple sequence repeats (SSRs) loci. The repeat thresholds for mono-, di-, tri-, tetra-, penta-, and hexa-nucleotide SSRs were set to a minimum of 10, 5, 4, 3, 3, and 3 repeats, respectively. The online program REPuter (Kurtz et al. 2001) was used to locate the large repeat sequences with a minimal repeat size of 30 bp and hamming distance equal to 3. In this study, 68 SSRs was found, including 38 mono-nucleotide SSRs, 16 di-nucleotide SSRs, four tri-nucleotide SSRs, 10 tetra-nucleotide SSRs. There were no penta- and hexa-nucleotide repeats. In addition, 31 large repeat sequences were identified, containing 11 forward repeats, 18 palindromic repeats, and two reverse repeats. These repeats ranged from 30 to 50 bp in length. SSRs and large repeat sequences can provide important information for research on population genetics and genetic markers.

In order to ascertain phylogenetic position of *L. martagon* var. *pilosiusculum*, a maximum likelihood (ML) phylogenetic tree was constructed with CIPRES (http://www.phylo.org/) (Miller et al. 2010). The complete chloroplast genome of 14 representative species from genera *Lilium* and *Cardiocrinum* (the genus *Cardiocrinum* species as outgroup) were selected to perform the ML analysis (Figure 1) (Kim and Kim 2013; Kim et al. 2015; Mennes et al. 2015; Bi et al. 2016; Du et al. 2016; Ji et al. 2016; Lu et al. 2016; Du et al. 2017; Zhang et al. 2017). The result indicated that 10 of 12 nodes were supported by bootstrap values 100% and the other two nodes by values >71%. In this ML tree, *L. martagon* var. *pilosiusculum* is recognized a species of *lilium* with a strong phylogenetic signal. Moreover, the species is closely related to *L. hansonii*, *L. tsingtauense*, *L. cernuum*, and *L. longiflorum* in this tree topology structure. The complete chloroplast genome can be subsequently utilized for genetic diversity, identifying species, taxonomy and phylogenetic evolution studies for this species. The available genome information also provides valuable insight into conservation and exploitation efforts for this endangered species.

**Figure 1.**
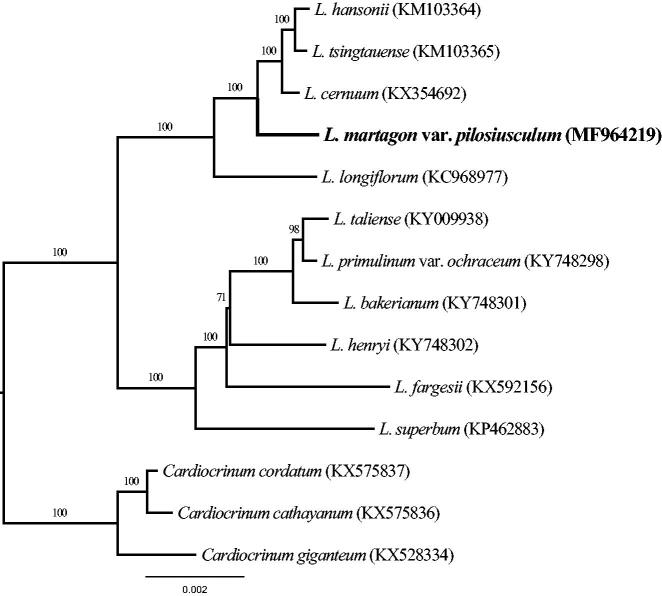
Phylogenetic relationships of 11 sequences in the genus *Lilium* with the outgroup of three *Cardiocrinum* species constructed by whole chloroplast genome with the maximum likelihood (ML) analyses. The bootstrap values were based on 1000 replicates.
